# Supplementary material for: Cellular crosstalk in atherosclerotic plaque microenvironment
Source: Cell Commun Signal. 2023 May 30;21:125. doi: 10.1186/s12964-023-01153-w (PMC10227997; doi:10.1186/s12964-023-01153-w)
Supplement: Supplementary file 2 — Additional file 1. [file 12964_2023_1153_MOESM1_ESM.docx]

**Title:**

**Cellular crosstalk in atherosclerotic plaque microenvironment**

**Running Title:**

**Atherosclerotic plaques**

**Authors:**

Elmira Mahdinia^1+^

Nafiseh Shokri^1+^

Abdolkarim Talebi Taheri^2^

Sahar Asgharzadeh^3^

Mohammad Elahimanesh^1^

Mohammad Najafi^1,4,**^

1 Department of Clinical Biochemistry, School of Medicine, Iran University of Medical Sciences, Tehran, Iran.

2 Department of Clinical Biochemistry, Faculty of Medicine, Shahid Beheshti University of Medical Sciences, Tehran, Iran.

3 Department of Clinical Biochemistry, Faculty of Medicine, Ghazvin University of Medical Sciences, Ghazvin, Iran.

4 Microbial Biotechnology Center, Faculty of Medicine, Iran University of Medical Sciences, Tehran, Iran.

**+ First Author**

Elmira Mahdinia and Nafiseh Shokri

****Corresponding author**

Mohammad Najafi, Department of Clinical Biochemistry, School of Medicine, Iran University of Medical Sciences, Tehran, Iran. [nbsmmsbn@iums.ac.ir](mailto:nbsmmsbn@iums.ac.ir)

**Abstract**

Atherosclerosis is an underlying pathology of many vascular diseases as a result of cellular, structural and molecular dysfunctions within the sub-endothelial space. This review deals with the events involved in the formation, growth and remodeling of plaque, including the cell recruitment, cell polarization, cell fat droplets. It also describes cross talking between endothelial cells, macrophages, and vascular smooth muscle cells, as well as the cellular pathways involved in plaque development in the plaque microenvironment. Finally, it describes the plaque structural components and the role of factors involved in the rupture and erosion of plaques in the vessel.

**Keywords:** Atherosclerosis, Polarization, Plaque, Rupture, Erosion

Atherosclerosis process develops due to many cellular and molecular events in the vessel. It is well known that during the progression of this process, the macrophages and vascular smooth muscle cells (VSMCs) polarize and together with extracellular matrix (ECM) components contribute to develop the primary core of atherosclerotic plaque in media. Furthermore, recruiting blood cells into sub-endothelial space and the remodeling plaque relate to microenvironment events. This review explains the principles of molecular and cellular events in the atherosclerosis process and signaling pathways involved in cellular polarization and cross talking in vessel sub-endothelial space.

1. **Recruiting blood cells into sub-endothelial space increases via diapedesis during atherosclerosis process**

Diapedesis is a process by which leukocytes pass through the vascular endothelium into the sub-endothelial space. It may occur in the paracellular or transcellular forms, known as inter- and intracellular ways, and includes cellular adhesion, movement, and migration [[1](#_ENREF_1)]. Many studies reported the roles of some genes in the paracellular and transcellular ways of diapedesis. The PECAM1 and CAV1 gene families are reported to transfer leukocytes through the vessel wall [[2](#_ENREF_2)] so that the downregulation of PECAM1 causes to the accumulation of leukocytes within the basement membrane [[3](#_ENREF_3), [4](#_ENREF_4)]. Some genes are also reported in the leukocyte trafficking pathway [2, [5](#_ENREF_5), 6]. These genes may be regulated by non-coding RNAs, including miRNAs [[7](#_ENREF_7)]. The adhesion molecules through their receptors, in addition to being involved in cell-cell interactions, can transduce the bidirectional signals between the endothelial cells and leukocytes causing to vascular permeability [8-[12](#_ENREF_12)]. Moreover, chemokines through the CXCR1 and CXCR2 receptors activate the integrins and adhesion molecules via G and beta-arrestin proteins [[13](#_ENREF_13), [14](#_ENREF_14)]. Duffy antigen receptor for chemokine (DARC), known for binding to CXC family, is responsible for delivering the chemokines by the endothelial cells. Furthermore, IL6 causes to express the adhesion molecules and some chemokines, such as CCL2 through GP130 in neutrophils [[14](#_ENREF_14)]. The blood cells also migrate via other receptor/ligand complexes during diapedesis process. The N-formyl-methionyl-leucyl-phenylalanine (FMLP) binds to formyl-peptide receptor (FPR) and causes cell migration [[14](#_ENREF_14)]. The expression of CD99 on endothelial cells is required for neutrophil trans-endothelial migration [[15](#_ENREF_15)]. Furthermore, the neutrophils and macrophages are involved in the inflammatory responses via FC (free cholesterol) receptor [[10](#_ENREF_10), 16]. Adenosine also increases neutrophil chemotaxis and phagocytosis, albeit at low concentrations, through the adenosine receptor subtypes [[17](#_ENREF_17)]. RAP1, RAP2 and Rho Family can regulate endothelial permeability and leukocyte trans-endothelial migration [[18](#_ENREF_18)]. The cellular permeability also increases by TNF-α through tight junctions (TJ), adherent junctions (AJ) and actin filaments. It is also increased by the histamine and bradykinin through VE-cadherin-related pathways [[18](#_ENREF_18)]. Adhesive leukocyte signals through GEF-small Rho GTPase axis quickly affect cell-cell connections. The effect of Rho, together with radial stress fiber, leads to the increased vascular permeability. The Rho-cross talked pathways also mediate actomyosin contraction led to cellular migration [[12](#_ENREF_12)]. Furthermore, the P-Rex/Rac signaling pathway increases vascular permeability by producing ROS compounds [[12](#_ENREF_12)]. The CD99 also facilitates the movement of leukocytes through TRPC6 calcium channels and adhesion molecules such as ICAM1, VCAM1 and PECAM1 on the endothelial cells [[11](#_ENREF_11), [19](#_ENREF_19), [20](#_ENREF_20)].

The leukocytes are the first line of blood cells in vascular permeability via the chemokine-followed inflammatory events [[8](#_ENREF_8), [21](#_ENREF_22)-[23](#_ENREF_23)]. Some diseases associated with chronic inflammation, such as lupus and psoriasis are highly exposed to cardiovascular diseases [[24](#_ENREF_24)]. When the inflammation begins, in the first stage, endothelial cells absorb leukocytes [[8](#_ENREF_8)] so that the cellular rolling occurs by adhesive reactions. During adhesion, cellular morphology is changed: first round, then flat, and finally rounded [[8](#_ENREF_8)]. Furthermore, the recruitment of leukocytes by activating memory T cells is followed by inflammatory cytokines [[23](#_ENREF_23)]. Moreover, chemical adsorbents when attach to their ligands, lead to the movement of leukocytes, a process that is called chemotaxis [[25](#_ENREF_25), [26](#_ENREF_26)]. The complementary components C3a and C5a can be involved in absorbing inflammatory cells [[26](#_ENREF_26), [27](#_ENREF_27)]. Other factors such as N-formyl peptides [[28](#_ENREF_28)], VWF, and WPB [[29](#_ENREF_29)] may cause vascular inflammation. It is well known that during inflammation, APO-A1 changes in HDL particles so that its decrease is associated with an increase in VWF [[29](#_ENREF_29)]. Also, cellular connections on endothelial cells change the permeability in response to a series of compounds. For example, a set of pro-inflammatory stimuli such as thrombin and histamine increase permeability while sphingosine 1-phosphate and angiopoietin-1 which act as anti-inflammatory agents, reduce cellular permeability [[18](#_ENREF_18)]. In vitro, the FMLF bacterial peptide binds to FPRs and causes neutrophil migration and polarization [[9](#_ENREF_9), [30](#_ENREF_30)]. Adenosine is also known an inflammatory modulator, so the adenosynthetic system is suggested as a therapeutic target [[31](#_ENREF_31)].

As indicated in the above, the neutrophils, leukocytes, and T cells migrate through activated arterial walls, which is a prerequisite step due to the infection or damage [[32](#_ENREF_32)]. The entry of cells into the inflamed locations occurs in several stages including the weak adhesion, rolling, and crawling between endothelial cells and leukocytes [[8](#_ENREF_8), [33](#_ENREF_33)] (Figure 1). The leukocyte adhesion cascade is sequentially performed by selectin and integrin on the cell surfaces [[32](#_ENREF_32)]. In addition to the endothelium, leukocytes must pass through the pericyte layer, the basement membrane of blood vessels. The neutrophils, however, do not pass through pericyte bodies but through pericyte gaps [[32](#_ENREF_32)]. In this way, IL1β increases the expression of several genes, including VCAM1, CX3CL1, MCP1 and IL6 in pericytes [[34](#_ENREF_34)]. Furthermore, the CXCL8 secretion from pericytes by IL1β, LPS and TNF-α progressed the transport of neutrophils[[32](#_ENREF_32)] . Moreover, pericytes express MHC2 when stimulated with cytokines, which increase phagocytosis capability of neutrophils [[32](#_ENREF_32)]. The polar shape that cells take on is necessary to cross the endothelial barrier. The areas where leukocytes leave the vessel , are called low expression regions (LERs), have low extracellular matrix proteins such as laminin 8, laminin 10, collagen IV and nidogen [[32](#_ENREF_32)]. The strong adhesion is mediated by a set of adhesion molecules in the immunoglobulin superfamily, including leukocyte integrins that bind to endothelial ligands such as ICAM1 and VCAM1. β2 Integrin is also essential for the transcellular movement of leukocytes. LFA1 and MAC1 are expressed by neutrophils and cause to adhere tightly and control cell crawling [[32](#_ENREF_32), [33](#_ENREF_33)]. It is also well known that during migration, actin filaments are responsible for the polymerization of the main edge of the cell, but actomyosin prevents protrusion in the lateral membrane [[8](#_ENREF_8)]. When leukocytes begin the transmigration process, the appearance of the leukocytes is rounder than their predecessor )crawling( just before they leave. Glycocalyx, at the apical surface of endothelial cells, immobilizes chemokines to promote integrin-induced adhesion [[21](#_ENREF_21)]. Platelets may be one of the modulators of inflammatory reactions in atherosclerosis via binding to endothelium by integrin and ICAM1 [[36](#_ENREF_36)]. As indicated in these studies, the endothelial function is impaired, the diapedesis is disrupted and blood cell outflow occurs excessively in the atherosclerosis process. These blood cells including monocytes can be polarized into macrophages and develop the atherosclerosis process in vessel sub-endothelial space.

1. **Macrophage polarization affects the atherosclerosis process**

The monocytes polarize to different macrophages in vessel sub-endothelial space. Some agents involved in the macrophage polarization, characteristics and their roles in the development of atherosclerosis are explained in the following. Atherosclerotic plaques contain mostly M1 and M2 macrophages. The microenvironment surrounding macrophages can acquire different phenotypes. For example, interferon γ and LPS activate the M1 macrophage [[37](#_ENREF_37)]. M0 macrophages can be converted to M1 and M2 macrophages by LPS/IFN-γ and IL4/IL-13 [[38](#_ENREF_38)]. The subgroups of M2 macrophages are known as M2b, M2c, M2a and M2d [[39-41](#_ENREF_39)]. M0 is polarized by IL4/IL13, LPS/IL1β and, IL10/TGF-β for the generations of M2a, M2b and, M2c macrophages, respectively [[42](#_ENREF_42)]. M0 macrophage is also polarized into M3 macrophages through TGF [[43](#_ENREF_43)] (Figure 2). M4 macrophages feature both M1 and M2 macrophages, and are induced by CXCL4 [[39](#_ENREF_39)]. In inflammatory conditions, macrophages form M1, while polarization to M2 occurs under anti-inflammatory conditions [[44](#_ENREF_44)]. M1 macrophages are abundant in areas prone to shoulder rupture, but M2 macrophages are found in the areas such as adventitia [[37](#_ENREF_37), [45](#_ENREF_45)]. M4 macrophages express proinflammatory chemokines such as TNF-α, IL6, MMP7 and MMP12. Furthermore, M4 macrophages have HLA–DR less than M1/M2 macrophages [[46](#_ENREF_46)] (Figure 3).

In atherosclerotic lesions, the macrophages scavenge ox-LDL particles and can convert into foam cells. In this state, the macrophages produce large amounts of ROS so that these compounds decrease M2 macrophage polarization [[47](#_ENREF_49), [48](#_ENREF_52)]. The polarization of macrophages towards M2 by mir-27a also occurred [[7](#_ENREF_7)]. The atherosclerotic plaque stability is associated to calcification and the kind of polarized macrophages in ruptured areas. The M2 macrophage is reported to eliminate inflammation while the M1 is involved in the progression of plaque, so it is proposed that the M2 macrophages play an important role in the prevention of inflammation by secreting IL-10 [[37](#_ENREF_37)]. M1 macrophages are seen in primary atherosclerotic lesions, while M2 macrophages are more in advanced lesions. M4 macrophages can also be seen in atherosclerotic lesions and cause the instability of fibrous cap in the plaque [[46](#_ENREF_46)].

The polarization of macrophages towards M1 macrophage is done by Th1 (T helper 1), TNF-α and GM-CSF [[49](#_ENREF_47)]. However, M1 macrophages have low IL10 values, but IL6 and IL1β produced by these macrophages are responsible for the progression of inflammation [[49](#_ENREF_47)]. Furthermore, TLR ligands (LPS) and other cytokines, such as IFN-γ are involved in the formation of M1 macrophages. CXCL4 cytokine leads to the polarization of monocytes to M4 macrophages [[41](#_ENREF_41), [46](#_ENREF_46)]. By treating M1 macrophages with IL4, or using IL13, the polarization can be converted to reveal anti-inflammatory phenotypes [[50](#_ENREF_48)]. When damage occurs, M1 macrophages begin to produce a variety of inflammatory molecules such as TNF-α, inducible nitric oxidase synthase (iNOS) and IL-12 [50]. LncRNA-COX2 is contributed to the elevation of iNOS, IL12 and TNF-α, which are more pronounced in M1 macrophages [[41](#_ENREF_41)]. M1 and M2 macrophages have different arginine metabolism; M1 macrophages, primarily through iNOS use arginine to produce nitric oxide, while M2 macrophages convert arginine to ornithine and urea through arginase [[51](#_ENREF_49)]. M2 macrophages can induce macroscopic calcium deposition by VSMC maturation and osteoblastic differentiation, called macrocalcification, which is associated with chronic inflammation [[37](#_ENREF_37)]. M2 macrophages have low levels of IL12 and IL23, however, they have high IL10 values. IL10, M-CSF and Th-2 cytokines stimulate the generation of M2 macrophages [[50](#_ENREF_47), [51](#_ENREF_48)]. M2 macrophages produce anti-inflammatory cytokines including IL4, IL5, IL10, IL13 and TGF-β [[49](#_ENREF_47), [50](#_ENREF_48)]. STAT1 mediates M1 macrophage activation, while STAT6 mediates M2 macrophage activation [[50](#_ENREF_48)]. STAT1 and STAT6 can inhibit each other; that is, when STAT6 is activated, it suppresses STAT1-dependent transcription and vice versa [[50](#_ENREF_48)]. IL4 can activate STAT6 and the cell phenotype moves towards M2 [[28](#_ENREF_28), [48](#_ENREF_48)]. However, STAT3 induces M2c Macrophage by IL-10 [[41](#_ENREF_41)]. MCP-1)monocyte chemotactic protein-1( is also reported as influential factors on macrophage polarization .[[7](#_ENREF_7)] M2b markers include IL10, CCL1, LIGHT, CD86, SPHK1, TNF-α, and IL6. To identify M2b, however, the IL10 marker is not suitable and other markers should be checked because all these M2 macrophages secrete IL10 abundantly. LncRNA GAS5 suppresses the CCL1 gene. The CCL1 is essential for the polarity of M2b macrophages [[41](#_ENREF_41)]. Also, miR-223 is considered to contribute for M2 polarization [[41](#_ENREF_41)]. Both M1 and M2b macrophages contain CD86, so this marker cannot distinguish M1 from M2b macrophages. However, it is useful to recognize M2b from other subclasses of M2 macrophages. TNF-α is also secreted by both M1 and M2b macrophages [[41](#_ENREF_41)].

The macrophage polarization relates mainly to the activation of some pathways such as PI3K/AKT, NF-KB, STAT1/STAT6 and MAPK/ERK [[51](#_ENREF_50)]. Adiponectin decreases the expression of some cytokines via the inhibition of NF-KB so that it directs M2 macrophages via AMPK and PPARα pathways. Adiponectin may also increase the secretion of other cytokines, such as TNF-α, IL6, and IL12. However, there were some controversies on the role of adiponectin as a pro-inflammatory or anti-inflammatory factor [[51](#_ENREF_49)]. MAPKs are involved in the polarization of M2b macrophages through the activation of ERK1/2, p38, and JNK. MBL, mannose-binding lectin, inhibits the signaling pathways related to MAPK and NF-KB, resulting in the decrease of polarity of M2b macrophages. Moreover, the polarization of M2b macrophages can be done by PI3k pathway [[41](#_ENREF_41)] (Figure 4). On the other hand, the polarization of M1 macrophages can be done by NOD-Like receptor related pathways [[52](#_ENREF_51)]. The P65 and P50 subunits of NF-κB can create pro-inflammatory and anti-inflammatory phenotypes in macrophages. When the NF-κB P65 subunit is activated, it promotes the polarization of M1 macrophages (Figure 5). The M2b macrophage is polarized by the activation of P50 subunit. NF-κB and IRF are activated by ALD-DNA (activated lymphocyte-derived DNA), which are involved in the polarization of macrophages towards M2b phenotype [[41](#_ENREF_41)]. M1 phenotype also occurs with INF-γ, which acts through the STAT1 pathway. The PI3K/AKT signaling pathway is activated by IL4 and its effect on the formation of macrophage phenotypes depends on its isoforms. The P110γ isoform causes the M1while the P110αβγ isoform makes M2 phenotype. AKT2 and AKT1 pathways are also involved in the M1 and M2 phenotypes, respectively. It also reported that the macrophage anti-inflammatory phenotype produced by IL4 is involved with MAPK/ERK signaling pathway [[51](#_ENREF_50)].

The macrophage population is originated from different cells and maintained by different agents in tissues. The conventional theory that each tissue macrophage originated from the bone marrow circulatory monocytes is changed by reporting that macrophages from embryonic progenitors may persist into maturity and self-maintain.  In a few cases, tissue-resident macrophages are completely embryo-derived, including microglia inside the brain, while others are continuously substituted from monocytes (LP) [[53](#_ENREF_53), [54](#_ENREF_54)]. Yet, most tissue-resident macrophages show a middle state of affairs and can be from combined origins [[55](#_ENREF_55)]. Nevertheless, monocytes additionally contribute to the resident macrophage population, on which the nearby environment may inflict tissue-specific macrophage functions. Each embryonic progenitor cell addition to monocytes can contribute to all populations, however, monocytes provide an upward push to MHCII+ greater effortlessly than to CX3CR1+ MHCII cardiac macrophages ensuing in a change of the resident macrophage pool permanently. Commonly, it is showed that monocytes and embryonic progenitors change their phenotypes into MHCII+ and CX3CR1-CMs rather than CX3CR1+ MHCII−[[56](#_ENREF_56)]. The cardiac macrophage (CM) pool is not stagnant, CMs of embryonic foundation are consecutively misplaced with age during postnatal growth. Resident embryo-derived mononuclear phagocytes may be changed under inflammatory or challenging conditions such as clodronate and angiotensin II [[56](#_ENREF_56)]. The microenvironment of organs can have an important role in the function of specific driven macrophage population. The tissue signals may define tissue-specific macrophages. In the resident mononuclear phagocyte of the peritoneum, for instance, vitamin A through GATA6 leads to cell polarization [[57](#_ENREF_57)]. Multiple cytokines were proven to have essential roles in cellular function. Macrophage-colony stimulating factor (M-CSF) is compulsory for development, survival, morphology, and function of macrophages. M-CSF significantly adjusts the macrophage population. Another essential cytokine involved in the adjustment of tissue macrophage turnover is granulocyte-macrophage colony-stimulating factor (GM-CSF). GM-CSF may induce the proliferation of mononuclear phagocytes and is necessary to keep the macrophages pool in a steady state [[58](#_ENREF_58)]. IL-4 has shown an extra key role in the regulation of mononuclear phagocyte proliferation and has proven to intercede the accumulation of pleural mononuclear phagocytes [[56](#_ENREF_56), [59](#_ENREF_59)]. Remarkably, the decrease in the overall proliferation rate of cardiac macrophages with age is associated with the decrease in the embryo-derived cardiac macrophages (51). The progressive loss of the tissue self-renewal capacity can result in the enhanced invasion of mononuclear phagocytes with decreased proliferative capacity, which lead to a gradually changed in subset combination of the cardiac macrophage pool. This indicates that monocyte recruitment can happen in inflammation and stress conditions, which can discharge the tissue population, and be a compensatory route to preserve macrophage homeostasis. Therefore, the ratio of ontogenetically differentiated macrophages to a tissue-resident pool can be dependent on their different self-renewal ability and microenvironment condition. Furthermore, the macrophages shift towards foam cells during atherosclerosis process that is related to their polarization

1. **Lipid deposition changes in macrophages during atherosclerosis process**

The low-density lipoprotein (LDL) values within the peripheral blood regulate by mononuclear phagocytes .In according to some hypothesizes suggested in atherosclerosis process, the macrophages absorb excessively modified lipoproteins via several LDL scavenger receptors (SR), such as SR-A1, CD36, and lectin-like ox-LDL receptor-1 (LOX-1) [[60](#_ENREF_61)]. Macrophages also express cholesterol transporters such as ABCA1, ABCG1, and SR-BI, which associate to reverse cholesterol transport (RCT) and are suggested as promising targets in cardiovascular disease [61]. In atherosclerosis, pro-inflammatory conditions increase the expression of scavenger receptors, particularly LOX-1, and decrease the expression of transporters associated with RCT. The internalized lipids are transferred to late endosomes/lysosomes and degrade by lysosomal enzymes such as lysosomal acid lipase (LAL) so that its defect is related to dyslipidemia [62]. Free cholesterol is successively processed into cholesteryl ester in cytosol with the aid of acetyl-CoA acetyltransferase (ACAT1), a contributing factor to macrophage foam cell death [[16](#_ENREF_16), [63](#_ENREF_63), [64](#_ENREF_64)]. The endoplasmic reticulum is a repository for retaining recent cholesteryl esters. It can be processed by neutral cholesterol ester hydrolase (nCEH), to produce free cholesterol that can be excreted through cholesterol transporters. Also, ACAT1 is overexpressed whereas nCEH expression is suppressed, so that it leads to the free and esterified cholesterol depositions in macrophages and finally the generation of foam cells [[65](#_ENREF_65)] (Figure 6). The lipid droplets (LDs) are storage particles in most cells. LDs are mainly located within the cytoplasm, however, the nucleus conjointly includes LDs in some cells [[66](#_ENREF_66)]. These lipid particles are also mainly composed of a neutral lipid core (oily phase), which consists of triacylglycerol (TG) and sterol esters. The organization and accommodation of neutral lipids in LDs are additionally necessary for safeguarding cells from lipotoxicity events, and the extra lipid prevention in cell membranes [[67](#_ENREF_67), [68](#_ENREF_68)]. LD facilitates the protein localization, which may include enzymes associated to LD metabolism, for example, some enzymes of neutral lipid biosynthesis and lipolysis pathways [[68](#_ENREF_68), [69](#_ENREF_69)]. In mammalian cells, LD biogenesis is related to the ER membrane phospholipids [[70](#_ENREF_70)-71]. **It** can be expedited through lipids that procreate an adequate flexion together with lysophospholipids. The very last notch of LDs from the ER can be supported by lipids that produce a poor flexion, together with palmitic acid (PA) and diacylglycerols (DAGs). Certainly, it had been mentioned that PA generated by phospholipase D (PLD) was essential for LD establishment [[72](#_ENREF_72)]. Moreover, RalA and PLD1 promoted lipid droplet growth [[73](#_ENREF_73)]. FIT2, a transmembrane protein of the endoplasmic reticulum, also establishes LDs and particularly, the route of LD sprouting [[74](#_ENREF_74)]. FIT2 can optimize the regional density of DAG on LD. DAG, which is manufactured by Pah1/Lipin, is needed for preliminary LD establishment. DAG may indicate the places of LD beginning from the endoplasmic reticulum. FIT2 might also manage the extent and trans-bilayer spatial property of DAG, controlling the route of LD germination. In the lack of FIT2, DAG collects on the cytosolic side of pre-LDs, which could enhance the monolayer tension and avert ordinary LD emergence into the cytosol [[72](#_ENREF_72)]. Seipin, an inimitable protein, adjusts the cell lipid reservoirs [[75](#_ENREF_75)]. Moreover, Seipin adjusts the positional biogenesis and dispensation of phospholipids on the endoplasmic reticulum [[72](#_ENREF_72), [76](#_ENREF_76)]. Some reports suggested a constructional function for seipin on the ER-LD interaction. The ER-LD interaction may also comfort the conduction of proteins and lipids to make certain regulators of LD growth, and can additionally simplify the cross-talks among ER-resident proteins and these on the LD floor. The ER-LD may be essential for the conduction of phospholipids to LD, resulting in the enlargement of LD [[72](#_ENREF_72), [77](#_ENREF_77), [78](#_ENREF_78)]. In cancer cells, LDs are a place of PGE2 (prostaglandin E2) biogenesis, a critical immunomodulator eicosanoid, and are related to tumor progression routes. In myeloid-derived cells, LDs had been related to the polarization of TAM (tumor-associated macrophage), a modified form of MDSCs (myeloid-derived suppressor cells), and in dendritic cells, LDs which have excessive oxidized triacylglycerol forms include a specific kind of antigen disruption. Based on some reports, LDs serve as a place for esterified arachidonic acid and engage with eicosanoid biogenesis enzymes such as cPLA2, cyclooxygenases and prostaglandin synthases [79].

Signaling routes entangled in lipid aggregation are widely recognized. CD36 has the important role in atherosclerosis pathophysiology. CD36 is a particular receptor to scavenge the ox-LDL particles, which cooperates in foam cell formation [[80](#_ENREF_79)]. The interplay between CD36 and ox-LDL elevates the phosphorylation of Src kinase, which prompts the Jun kinase (JNK 1 and 2) and also VAV scavenging of ox-LDL. A report suggested that the lipid accumulation in macrophages is upraised via two ways; Wnt5a, which is enabled by Fz5 signaling pathway and through PPARγ, which is activated by p38 MAPK signaling pathway [[81](#_ENREF_80)]. TLR2 turns on by CD36, co-expresses with Wnt5a, and cooperates in foam cell formation. TLR4 can also be involved in lipid repletion through PI3K/mTORC2 route, followed by the AKT phosphorylation [[81](#_ENREF_80), [82](#_ENREF_81)]. The mTOR signaling route is proven to be stimulated during foam cell formation. The stimulated endothelial NF-κB route raises macrophage function in atherosclerotic plaques. Further particularly, macrophage-produced foam cells can spatter TNF-α and different cytokines to utilize immune cells, cooperating with the formation of plaques and growing the danger of cardiovascular events [[83](#_ENREF_82)]. Whilst extra naive lipids, which include fatty acids and sterols, collect within the ER. Some cellular pathways suggest to convert these lipids to greater inert neutral lipids. For example, the ACAT produces sterol esters, and DGAT produces TGs and different neutral lipids to be accommodated in LDs [[68](#_ENREF_68), 84]. Each disturbance in the lipid droplet synthesis pathway may cause to deposit the lipid in macrophages and be converted to the foam cells so that these cells may produce the initial core of atherosclerosis plaques.

1. **Macrophages and VSMCs are involved in plaque remodeling during the development of atherosclerosis process**

Atherosclerosis plagues are initially produced due to aggregate the foam cells, yellow xanthomas cells, in the intima proteoglycan layer. When necrosis occurs in foam cells, the necrotic nucleus develops and the connective components substitute gradually with collagen-rich materials in the primary core. Then, it develops by adding other cells such as VSMCs, macrophages and ECM compounds. Vulnerable plaques include a necrotic nucleus that occupies 30% of the plaque and a fibrous cap whose thickness is to be estimated as less than 65 micrometers. The fibrous cap is located between the vascular lumen and the necrotic nucleus [[85](#_ENREF_85)] so that it may be related to angiotensin II and TGF-β released from VSMCs [[86](#_ENREF_86)]. The apoptotic macrophages are found at the sites of plaque rupture. It is reported that these macrophages express the cytokines that over-proliferate VSMCs [[87](#_ENREF_87)]. It is also known that matrix metalloproteinase-9 (MMP-9) produced by macrophages causes plaque rupture [[88](#_ENREF_88)]. Moreover, VSMCs and collagen are reported to be in fibrous cap [[89](#_ENREF_89)] while little collagen but abundant free cholesterol are observed in the necrotic nucleus of the plaque. SP1, SP3 and AP1 transcription factors interacting with the collagen promoter lead to the transcription of type I collagen in the cells [[86](#_ENREF_86)]. The absence of collagen is a sign of the loss of VSMCs that makes the plaque to be vulnerable [[85](#_ENREF_85)]. One of the reasons for impaired collagen deposition can be the reduction of metalloproteinase [[90](#_ENREF_90)]. The tensile strength of the fibrous cap is facilitated by collagen. When VSMCs are removed from the cap, it becomes thin, so that the ruptured cap has a small amount of collagen [[88](#_ENREF_88)]. If VSMCs over-reproduce, it leads to the growth of plaque dependent on angiogenic factors [91, 92]. Type VIII collagen is in small amounts in normal arteries and produces by macrophages and VSMCs. It increases the migration and growth of VSMCs through the extracellular matrix. ApoE, located in HDL, suppresses the expression of type VIII collagen, so that its decrease is associated with an increase in type VIII collagen [[87](#_ENREF_87)]. HDL also reduces ox-LDL and TNF-α levels, and results in the reduction of endothelial cell apoptosis [[93](#_ENREF_93), [94](#_ENREF_94)]. The macrocalcification also increases plaque consistency, while microcalcification makes plaque vulnerable. The microcalcification is reported due to pro-inflammatory events in M1 macrophages to make spotty calcification in the necrotic nucleus [[37](#_ENREF_37)].

The roles of VSMCs in the formation and development atherosclerotic plaques are related to their phenotypes to be able to proliferate and migrate. The epithelioid VSMCs, a synthetic phenotype, have a cubic appearance, are present in small amounts in the arteries, and involve in the development of plaque. Another important VSMC phenotype in plaques is known as VSMC-derived foam cells made due to the accumulation of lipids in their cytosol [[94](#_ENREF_94)]. These cells have the high amounts of rough endoplasmic reticulum and Golgi apparatus, and their microfilaments are scattered [[88](#_ENREF_88)]. Their motility comprises four tandem steps: i, polarizing the cell. ii, spreading the lamellipodia at the edge of cell. iii, connecting the cell iv, and eventually contracting of the cell [[94](#_ENREF_94)]. If VSMCs go into apoptosis, the plaque rupture occurs [[96](#_ENREF_96)]. During cell phenotype changes, some signaling pathways are modified. Serine/threonine kinase is important in the cardiovascular system and its absence decreases the migration of VSMC cells. Furthermore, the proliferation and migration of VSMCs are inhibited by regulatory molecules [[95](#_ENREF_95)]. In addition, studies have shown that with the reduction of AKT, there was a reduction in the VSMC migration and instability of plaques [[96](#_ENREF_96)]. However, the phosphorylation of HCK protein increased the proliferation of VSMCs [80].

Addition to VSMCs, the phenotype of other cells such as macrophages affects the contents and stability of atherosclerotic plaques. M1 Macrophage produces the pro-inflammatory cytokines such as IL-6, IL-1, IL-23, IL-1β, TNF-α, IL-12 and IL-18, and changes the plaque microenvironment [[97-](#_ENREF_98)[101](#_ENREF_101)]. It also causes angiogenesis, releases the reactive oxygen/nitrogen species, and stimulates the early stages of tissue repair, tumor resection, increased glycolysis and fatty acid synthesis [[50](#_ENREF_50), [97](#_ENREF_97), [98](#_ENREF_98), [100](#_ENREF_100), [102](#_ENREF_102)]. 40% of all macrophages in atherosclerosis plaques are M1, mostly seen in advanced atherosclerotic lesions and in the shoulder area of atherosclerotic plaques (prone to rupture). The number of M1 macrophages increases with the progression of plaque [[37](#_ENREF_37), [50](#_ENREF_50), [97](#_ENREF_97), [100](#_ENREF_100)]. On the other hand, M2 macrophage, an anti-inflammatory cell, secretes the high levels of IL-10, CD163, CD206 and FIZZ1 [[98](#_ENREF_98), [103](#_ENREF_103)-[106](#_ENREF_104)]. M2 macrophages are involved in angiogenesis, tissue repair, wound healing, parasite inhibition, tumor progression, allergic inflammation, ossification, oxidation of fatty acids and reduction of glycolysis [[50](#_ENREF_50), [98](#_ENREF_98), [100](#_ENREF_100)]. 20% of all macrophages in atherosclerotic plaques are M2, mostly seen in the primary lesions of atherosclerosis and in the adventitia of atherosclerotic plaques [[97](#_ENREF_97), [100](#_ENREF_100), [101](#_ENREF_101)]. Their numbers decrease with the progression of plaque and causes plaque instability. M2 macrophages also change extracellular matrix in the final stages of the plaque healing process [[37](#_ENREF_37), [50](#_ENREF_50), [107](#_ENREF_107), 108]. These reports suggested that the progression rate of atherosclerosis process is related to the concurrent functions of cells in the plaque microenvironment.

1. **Adverse cellular crosstalk causes to enlarge atherosclerosis plaque**

The atherosclerosis plaque consists of fat deposits, cells (VSMCs, inflammatory cells, macrophages and endothelial cells), ECM, cytokines, and chemokines [[109-112](#_ENREF_109)]. The necrotic core is covered with a layer of type 1 collagen-rich matrix that originates from smooth muscle cells [[88](#_ENREF_88)]. The changes of phenotype of endothelial cells, macrophages and VSMCs not only affect their cellular functions but also cause some cell autocrine and paracrine effects to exacerbate the plaque microenvironment. When the laminar flow in the arteries is not continued and significant pressure is on the vessel wall, it disrupts endothelial function, led to changes in the cellular permeability of vessels, changes in the compositions of the extracellular matrix, and the entrance of low-density lipoprotein (LDL) particles [[113-115](#_ENREF_113)]. The disrupted endothelial cells cause to recruit the monocytes via the induced adhesion molecules on their membrane levels [[115](#_ENREF_115)] (Figure 7). The LDL particles oxidize by myeloperoxidase derived from inflammatory cells and cause to express CD36, TLR4 and TLR6 receptors on macrophages. Foam cells facilitate the migration of vascular smooth muscle cells (VSMCs) into the intima by secreting cytokines and matrix metallopeptidases (MMPs) [[113](#_ENREF_113), [115](#_ENREF_115)]. In intima, the macrophage cytokines such as IL6 and TNF-α change the phenotype of vascular smooth muscle cells from contractile to synthetic state causing the synthesis of collagen and elastin, which form the fibrous cap of plaque. Furthermore, these synthetic VSMCs can be located on a set of foam cells that their death due to necrosis causes to release and accumulate lipids. The accumulation of cellular debris and extracellular fat due to dysfunctional “efferocytosis” (cleansing of dead cells) by macrophages results in the formation of a fat-rich pool called the necrotic nucleus of plaque [[113](#_ENREF_113), [115](#_ENREF_115)]. In the sub-endothelium, excess cholesterol can also accumulate and cause the immune reactions due to oxidative and enzymatic modifications, which eventually creates an inflamed environment for the abnormal function of cells [[116](#_ENREF_116)].

The instability of plaques are also involved with various cellular events due to the mutual effects of proinflammatory, procoagulation and proteolytic molecules on the macrophages, smooth muscle cells, and endothelial cells [[117](#_ENREF_117)]. Changing the cell phenotype, which has the proinflammatory and prothrombotic state, is called cell activation, to be a risk factor for plaque remodeling. For example, the activation of endothelial cells and the accumulation of apoB-lipoproteins (Apo-B LPs) cause the movement of monocytes into the sub-endothelial space [[118](#_ENREF_118)]. ECs are also activated by various physiological and pathological changes, including disturbed blood flow dynamics [[119](#_ENREF_119)]. High one-way laminar shear stress (LSS), by positively regulating KLF2 transcription factor (Krüppel-like factor 2), maintains vascular unity by creating an anti-inflammatory and anti-thrombotic phenotype. KLF2 also inhibits glucose entry into the cells, suppressing glycolysis and mitochondrial respiration [[120](#_ENREF_120), [121](#_ENREF_121)]. While atherosclerotic susceptible areas are exposed to low LSS, ECs activate proinflammatory pathways, increasing glucose uptake, followed by increased glycolysis via NF-κB/HIF1α pathway [[122](#_ENREF_122), [123](#_ENREF_123)]. The primary binding of monocytes with the activated ECs is through chemokines, adhesion molecules and their ligands. The selectin family especially P-selectin is expressed by ECs and binds to P-selectin glycoprotein ligand-1 (PSGL-1) on monocytes [[124](#_ENREF_124), [125](#_ENREF_125)]. The attachment of leukocytes with the endothelium is related to impaired endothelial nitric oxide synthase (eNOS). NO is a vasodilator, and its reduction leads to the upregulation of vascular cell adhesion molecule 1 (VCAM-1), intercellular adhesion molecule 1 (ICAM-1) and E-selectin [[125](#_ENREF_125)]. Furthermore, the EC-leukocyte attachment may be improved through integrin family and their ligands [[125](#_ENREF_125)].

With the lipid consumption by macrophages, scavenger receptors induced and involved in the fat uptake are commonly including; sweeper receptor type A (SR-A), CD36 as a member of the type B family, LDL receptor-related protein 1 (LRP1) and lectin-like ox-LDL receptor 1 (LOX1) [126-[128](#_ENREF_127)]. In addition to conversion to foam cells, the macrophage secretions reduce collagen synthesis by VSMCs, thereby thinning the fibrous plaque cap so that the plaque becomes unstable [[124](#_ENREF_124)]. The unstable plaque disconnects from the ECs and moves through the artery. It, in turn, causes blockage in the lumen of susceptible arteries, and thromboembolic events such as heart attack or stroke [[124](#_ENREF_124)]. VSMCs have several phenotypes based on the compounds produced by macrophages and endothelial cells, including macrophage-like, contractile, synthetic, myofibroblast-like and necroptotic phenotypes. The macrophage-like and myofibroblast-like phenotypes have different functions on the vulnerability and stability of plaques. The macrophage-like cells increase the growth and disintegration of the necrotic core in plaque following the increased ox-LDL uptake and forming a very lipid-rich state. In contrast, the myofibroblast-like form by increasing the production of collagen leads to an increase in the diameter of the fibrous cap and, consequently, the increase of plaque resistance to rupture under the influence of various factors, including mechanical tension making the plaque stronger. Therefore, the VSMCs with the myofibroblast-like phenotype will play an important role in reducing the necrotic core (NC) size and thickening the fibrous cap of the plaque. It is proposed that in a stable plaque, the thickness of NC decreases up to 50% and in contrast, the thickness of the fibrous cap increases up to 4 times. The VSMC phenotypes, in addition to their roles in plaque morphology as mentioned above, are also involved in the changes of the plaque microenvironment [[95](#_ENREF_95), [129](#_ENREF_129)]. Another critical role for VSMCs is related to their ability to express different receptors for fat uptake, which, following this uptake, form foam cells, like macrophages, and ultimately increase the initial accumulation of fat in plaque [[129-131](#_ENREF_129)]. VSMCs also play an essential role in the onset and progression of inflammation through the secretion of cytokines such as PDGF, TGF-β, IFN-γ and MCP-1 so that by cascading reactions in other cells, they form fat streaks, followed by plaque progression [[132](#_ENREF_132), [133](#_ENREF_133)]. VSMCs are also involved in the expression of adhesion molecules, including VCAM-1 and ICAM-1. The adhesion molecules increase the resistance of VSMC cells to apoptosis and abnormal proliferation progressing the number of cells present in the lesion in the early stages [[132](#_ENREF_132)]. In more advanced settings, VSMC apoptosis may have adverse effects such as plaque prone to rupture via thinning of the fibrous cap, the increased necrotic core and loss of structural proteins [[134](#_ENREF_134)]. In general, it has been shown that in advanced lesions, one of the important factors for plaque stability and growth is abnormal proliferation by VSMCs in cooperation with matrix deposition so they affect the fibrous cap [[92](#_ENREF_92)]. These events ultimately lead to the formation of ruptured plaques [[133](#_ENREF_133), [135](#_ENREF_135)]. In ruptured plaques, cigarette smoke and Polycyclic aromatic hydrocarbons lead to a pathological thrombotic environment by activating and accumulating platelets, increasing platelet volume and circulation, increasing plasma fibrinogen, increasing clot strength, stimulating coagulation cascade and disrupting fibrinolysis [[135](#_ENREF_133), [136](#_ENREF_135)]. The studies, however, showed that the plaques form due to the cell dysfunctions in vessel sub-endothelial microenvironment but the fate and effect of plaques on the vessel microanatomy relate to their components.

1. **Plaque content relate to its stability**

The plaque stability relates to the density of cells, internal necrosis, angiogenesis, permeable endothelium and plaque bleeding. Intra-plaque hemorrhage, which occurs more frequently in fibroatheroma, is due to the enlargement of necrotic core. The size of necrotic core is essential for plaque stability, and its growth can decrease the fibrous cap and puts more tensile stress on the fibrous cap. A ruptured fibrous cap is also an important source of plaque bleeding. Neovascularization, which is accompanied by vasa vasorum, is permeable to plasma proteins and RBCs, and may leads to the plaque hemorrhage [[88](#_ENREF_88)]. Hypoxic inducers and growth factors are the regulatory mechanisms of plaque angiogenesis. The gradual transformation a lipid-rich plaque into a more fibrotic and calcific form stabilizes it [[103](#_ENREF_103), [107](#_ENREF_107)]. However, the balance between the fibrous cap and the necrotic core is essential for plaque stability [137]. The stable plaques have a thick fibrous cap, covered with inflammatory cells scattered on a small fat core, the rich in collagen, while the unstable plaques have a thin fibrous cap on a large fat nucleus [[115](#_ENREF_115), [116](#_ENREF_116), 138]. Decorin and biglycan proteoglycans provide tensile strength, thus stabilizing mature plaques [[107](#_ENREF_107), [137](#_ENREF_137), 139].

Plaque rupture occurs when the resistance of plaque disrupts by stress. It depends on some the plaque characteristics, for example the amounts of collagen and thrombogenic fats [[114](#_ENREF_114)]. Plaque rupture occurs more frequently in areas where the fibrous cap diameter is less than 65µm, platelets are active, inflammatory and foam cells are more permeable [[107](#_ENREF_107)]. M1 macrophages are abundant in areas prone to rupture the unstable plaques [[97](#_ENREF_97)]. In the ruptured plaques, the smooth muscle cells and ECM are reduced, and platelet-rich blood clots are produced [[137](#_ENREF_137)]. Proinflammatory cytokines reduce collagen synthesis and destroy the biomechanical integrity of plaque fibrous cap by increasing the overexpression of collagenases in plaque microenvironment [140]. The cytokines also increase the expression of potent procoagulant tissue factors in ruptured plaques and cause plaque thrombosis [[141](#_ENREF_139)]. The ruptured plaques come in contact with collagen, lipids and smooth muscle cells so that the coagulation and thrombosis processes activate by platelets [[115](#_ENREF_115), 142]. Thrombus has a fibrin rich red feature [[141](#_ENREF_139)]. In some cases, the plaque rupture and thrombosis occur spontaneously. The plaque rupture may also increase due to stress and hemodynamic changes [[137](#_ENREF_137)]. Most ruptures have no obvious clinical symptoms and only have thrombus on the vessel wall so that cause gradual narrowing of the coronary arteries. Moreover, the ruptures are more occurred in men (76%) as compared to women (55%). It is more common in postmenopausal women [[88](#_ENREF_88)]. A larger necrotic core increases the thrombogenic nature of plaque material and the risk of plaque rupture. On the other hand, thin cap fibroatheroma (TCFAs, intensification of mechanical stress on the plaque surface) accumulate in the vicinity of the main coronary arteries and increase the risk of plaque rupture [[88](#_ENREF_88)]. In ruptured plaque, small dense low-density lipoprotein cholesterol and triglyceride-rich lipoproteins are increased [143], HDL cholesterol is low, and the ratio of total cholesterol to HDL cholesterol is higher than the eroded plaques [[144](#_ENREF_140)]. To strengthen the fibrous cap of plaques and to reduce the risk of rupture, some lipid-lowering therapies are reported that reduce the inflammation and accumulation of fat in plaque [[141](#_ENREF_139)]. Moreover, the stenosis severity is related to the number of ruptured plaques [[88](#_ENREF_88)].

The plaque erosion occurs due to loss of antithrombotic events of the plaque surface, endothelial damage and excessive thrombosis in the absence of cap rupture. TLR2 is reported to alter the endothelial function and cause superficial erosion [[107](#_ENREF_107), [141](#_ENREF_139)]. Coronary vasospasm is one of the effective factors in the erosion pathophysiology [[144](#_ENREF_140)]. The plaque erosion exacerbates thickening fibroatheromas, the mechanism which is still unknown [[88](#_ENREF_88)]. Some eroded plaques have a multi-layered structure [[88](#_ENREF_88)]. Furthermore, they have a lot of VSMCs, ECM and a small accumulation of fat and foam cells [[141](#_ENREF_139)]. Inflammatory cells usually do not penetrate within it [[103](#_ENREF_103), [107](#_ENREF_107), [144](#_ENREF_140)] .In eroded plaques, there is an abundance of type III collagen, versican, and hyaluronan. Hyaluronan can directly increase fibrin polymerization and VSMCs migration towards plaque [[144](#_ENREF_140)] .In eroded plaques, the internal and external lamina layers remain untouched. Lack of endothelial lining causes these plaques to be in direct contact with the intima. The eroded plaque lesions are rarely calcified [[144](#_ENREF_140)]. In women under 50 years, more than 80% of thrombosis occurs due to plaque erosion, and the thrombi are white and platelet-rich [[141](#_ENREF_139), [144](#_ENREF_140)]. However, thrombosis is less likely to occur in the plaque erosion, but if it does happen, it is more fatal than thrombosis caused by the rupture. Moreover, thrombosis heals in ruptured plaques faster than in the eroded plaques [[88](#_ENREF_88), [144](#_ENREF_140)]. If plaque erosion be asymptomatic, it can cause plaque growth and gradual stenosis of the coronary artery. The eroded plaques are a cause of sudden cardiac death [[145](#_ENREF_88)] (Figure 8). In addition, intra-myocardial microembolism is also more likely to happen in the eroded plaques [[144](#_ENREF_140)].

**In conclusion**

Atherosclerosis is a multi-focal, slowly progressive process. It occurs due to numerous pathological changes including the disrupted cell and molecular functions in vessel sub-endothelial space [146-148]. The atherosclerotic plaques include cholesterol-rich core and fibrous cap. Furthermore, the acquired and innate immune agents cause to progress the plaque [[149](#_ENREF_144)-151]. Narrowing of arteries caused by the erosion and rupture of plaques slows down blood flow and results in fatal ischemia in the vessels [[152](#_ENREF_147)-155]. Thus, knowing the molecular changes and the cellular events in sub-endothelial microenvironment during the plaque growth can help to understand the stenosis in the vessels.

**Declarations :**

**Ethical Approval**
Not applicable.
**Competing interests**
The authors declare that they have no competing interests.
**Authors' contributions**
MN designed the study. EM, NS and ATT searched and prepared initial data. SA prepared the Figures. ME helped in revision.

**Funding**
Not applicable.
**Availability of data and materials**
Not applicable.

**References**

1. Schoppmeyer, R. and J.D. van Buul, *The diapedesis synapse: dynamic leukocyte-endothelium interactions.* Current Opinion in Physiology, 2021. **19**: p. 1-9.

2. Mickael, M.E., et al., *Paracellular and Transcellular Leukocytes Diapedesis Are Divergent but Interconnected Evolutionary Events.* Genes (Basel), 2021. **12**(2).

3. Sun, J., et al., *Platelet endothelial cell adhesion molecule-1 (PECAM-1) homophilic adhesion is mediated by immunoglobulin-like domains 1 and 2 and depends on the cytoplasmic domain and the level of surface expression.* J Biol Chem, 1996. **271**(31): p. 18561-70.

4. Hahn, J.H., et al., *CD99 (MIC2) regulates the LFA-1/ICAM-1-mediated adhesion of lymphocytes, and its gene encodes both positive and negative regulators of cellular adhesion.* J Immunol, 1997. **159**(5): p. 2250-8.

5. Gerhardt, T. and K. Ley, *Monocyte trafficking across the vessel wall.* Cardiovasc Res, 2015. **107**(3): p. 321-30.

6. Tuomisto, T.T., et al., *Analysis of gene and protein expression during monocyte-macrophage differentiation and cholesterol loading--cDNA and protein array study.* Atherosclerosis, 2005. **180**(2): p. 283-91.

7. Shaikhnia, F., et al., *miR-27a inhibits molecular adhesion between monocytes and human umbilical vein endothelial cells; systemic approach.* BMC Res Notes, 2022. **15**(1): p. 31.

8. Filippi, M.D., *Mechanism of Diapedesis: Importance of the Transcellular Route.* Adv Immunol, 2016. **129**: p. 25-53.

9. Mócsai, A., B. Walzog, and C.A. Lowell, *Intracellular signalling during neutrophil recruitment.* Cardiovasc Res, 2015. **107**(3): p. 373-85.

10. Futosi, K., S. Fodor, and A. Mócsai, *Neutrophil cell surface receptors and their intracellular signal transduction pathways.* Int Immunopharmacol, 2013. **17**(3): p. 638-50.

11. Poursaleh, A., et al., *Isolation of intimal endothelial cells from the human thoracic aorta: Study protocol.* Med J Islam Repub Iran, 2019. **33**: p. 51.

12. van Buul, J.D. and I. Timmerman, *Small Rho GTPase-mediated actin dynamics at endothelial adherens junctions.* Small GTPases, 2016. **7**(1): p. 21-31.

13. Rajarathnam, K., et al., *How do chemokines navigate neutrophils to the target site: Dissecting the structural mechanisms and signaling pathways.* Cell Signal, 2019. **54**: p. 69-80.

14. Kolaczkowska, E. and P. Kubes, *Neutrophil recruitment and function in health and inflammation.* Nat Rev Immunol, 2013. **13**(3): p. 159-75.

15. Muller, W.A., *Transendothelial migration: unifying principles from the endothelial perspective.* Immunol Rev, 2016. **273**(1): p. 61-75.

16. Yu, X.H., et al., *Foam cells in atherosclerosis.* Clin Chim Acta, 2013. **424**: p. 245-52.

17. Barletta, K.E., K. Ley, and B. Mehrad, *Regulation of neutrophil function by adenosine.* Arterioscler Thromb Vasc Biol, 2012. **32**(4): p. 856-64.

18. Cerutti, C. and A.J. Ridley, *Endothelial cell-cell adhesion and signaling.* Exp Cell Res, 2017. **358**(1): p. 31-38.

19. Pasello, M., M.C. Manara, and K. Scotlandi, *CD99 at the crossroads of physiology and pathology.* J Cell Commun Signal, 2018. **12**(1): p. 55-68.

20. Muller, W.A., *Localized signals that regulate transendothelial migration.* Curr Opin Immunol, 2016. **38**: p. 24-9.

21. Schimmel, L., N. Heemskerk, and J.D. van Buul, *Leukocyte transendothelial migration: A local affair.* Small GTPases, 2017. **8**(1): p. 1-15.

22. Leick, M., et al., *Leukocyte recruitment in inflammation: basic concepts and new mechanistic insights based on new models and microscopic imaging technologies.* Cell Tissue Res, 2014. **355**(3): p. 647-56.

23. Nourshargh, S. and R. Alon, *Leukocyte migration into inflamed tissues.* Immunity, 2014. **41**(5): p. 694-707.

24. Feingold, K.R. and C. Grunfeld, *The Effect of Inflammation and Infection on Lipids and Lipoproteins*, in *Endotext*, K.R. Feingold, et al., Editors. 2000, MDText.com, Inc.Copyright © 2000-2022, MDText.com, Inc.: South Dartmouth (MA).

25. Weiss, D. and B. Walcheck, *Neutrophil Function*. 2008. p. 331-350.

26. McDonald, B. and P. Kubes, *Chemokines: sirens of neutrophil recruitment-but is it just one song?* Immunity, 2010. **33**(2): p. 148-9.

27. Guo, R.F. and P.A. Ward, *Role of C5a in inflammatory responses.* Annu Rev Immunol, 2005. **23**: p. 821-52.

28. Dorward, D.A., et al., *The role of formylated peptides and formyl peptide receptor 1 in governing neutrophil function during acute inflammation.* Am J Pathol, 2015. **185**(5): p. 1172-84.

29. Chen, J. and D.W. Chung, *Inflammation, von Willebrand factor, and ADAMTS13.* Blood, 2018. **132**(2): p. 141-147.

30. Gambardella, L. and S. Vermeren, *Molecular players in neutrophil chemotaxis--focus on PI3K and small GTPases.* J Leukoc Biol, 2013. **94**(4): p. 603-12.

31. Pasquini, S., et al., *Adenosine and Inflammation: Here, There and Everywhere.* Int J Mol Sci, 2021. **22**(14).

32. Rudziak, P., C. Ellis, and P. Kowalewska, *Role and Molecular Mechanisms of Pericytes in Regulation of Leukocyte Diapedesis in Inflamed Tissues.* Mediators of Inflammation, 2019. **2019**: p. 1-9.

33. Poursaleh, A., et al., *Adhesion of monocytes and endothelial cells isolated from the human aorta suppresses by miRNA-PEI particles.* BMC Cardiovasc Disord, 2021. **21**(1): p. 395.

34. Rustenhoven, J., et al., *TGF-beta1 regulates human brain pericyte inflammatory processes involved in neurovasculature function.* J Neuroinflammation, 2016. **13**: p. 37.

35. Sluiter, T.J., et al., *Endothelial Barrier Function and Leukocyte Transmigration in Atherosclerosis.* Biomedicines, 2021. **9**(4).

36. Coenen, D.M., T.G. Mastenbroek, and J. Cosemans, *Platelet interaction with activated endothelium: mechanistic insights from microfluidics.* Blood, 2017. **130**(26): p. 2819-2828.

37. Shioi, A. and Y. Ikari, *Plaque Calcification During Atherosclerosis Progression and Regression.* J Atheroscler Thromb, 2018. **25**(4): p. 294-303.

38. Lee, K.Y., *M1 and M2 polarization of macrophages: a mini-review.* 2019.

39. Domschke, G. and C.A. Gleissner, *CXCL4-induced macrophages in human atherosclerosis.* Cytokine, 2019. **122**: p. 154141.

40. Hosseini-Fard, S., et al., *ATF3 and EGR2 gene expression levels in sdLDL-treated macrophages of patients with coronary artery stenosis.* LaboratoriumsMedizin, 2018. **12**.

41. Wang, L.X., et al., *M2b macrophage polarization and its roles in diseases.* J Leukoc Biol, 2019. **106**(2): p. 345-358.

42. Orekhov, A.N., et al., *Monocyte differentiation and macrophage polarization.* Vessel Plus, 2019. **3**: p. 10.

43. Li, Y., et al., *Role of Macrophages in the Progression and Regression of Vascular Calcification.* Front Pharmacol, 2020. **11**: p. 661.

44. Dolmatova, L.S. and I.Y. Dolmatov, *Different Macrophage Type Triggering as Target of the Action of Biologically Active Substances from Marine Invertebrates.* Mar Drugs, 2020. **18**(1).

45. Hassanpour, P., et al., *Interleukin 6 may be related to indoleamine 2,3-dioxygense function in M2 macrophages treated with small dense LDL particles.* Gene, 2017. **626**: p. 442-446.

46. Ruytinx, P., et al., *Chemokine-Induced Macrophage Polarization in Inflammatory Conditions.* Front Immunol, 2018. **9**: p. 1930.

47. Thapa, B. and K. Lee, *Metabolic influence on macrophage polarization and pathogenesis.* BMB Rep, 2019. **52**(6): p. 360-372.

48. Najafi, M., A. Roustazadeh, and B. Alipoor, *Ox-LDL Particles: Modified Components, Cellular Uptake, Biological Roles and Clinical Assessments.* Cardiovasc Hematol Disord Drug Targets, 2011. **11**(2): p. 119-28.

49. Businaro, R., et al., *Cellular and molecular players in the atherosclerotic plaque progression.* Ann N Y Acad Sci, 2012. **1262**: p. 134-41.

50. Ye, J., et al., *Promoting musculoskeletal system soft tissue regeneration by biomaterial-mediated modulation of macrophage polarization.* Bioact Mater, 2021. **6**(11): p. 4096-4109.

51. Nagenborg, J., et al., *Heterogeneity of atherosclerotic plaque macrophage origin, phenotype and functions: Implications for treatment.* European Journal of Pharmacology, 2017. **816**: p. 14-24.

52. Leitinger, N. and I.G. Schulman, *Phenotypic polarization of macrophages in atherosclerosis.* Arterioscler Thromb Vasc Biol, 2013. **33**(6): p. 1120-6.

53. Bain, C.C., et al., *Resident and pro-inflammatory macrophages in the colon represent alternative context-dependent fates of the same Ly6Chi monocyte precursors.* Mucosal Immunol, 2013. **6**(3): p. 498-510.

54. Tamoutounour, S., et al., *Origins and functional specialization of macrophages and of conventional and monocyte-derived dendritic cells in mouse skin.* Immunity, 2013. **39**(5): p. 925-38.

55. Sieweke, M.H. and J.E. Allen, *Beyond stem cells: self-renewal of differentiated macrophages.* Science, 2013. **342**(6161): p. 1242974.

56. Gentek, R., K. Molawi, and M.H. Sieweke, *Tissue macrophage identity and self-renewal.* Immunol Rev, 2014. **262**(1): p. 56-73.

57. Okabe, Y. and R. Medzhitov, *Tissue-specific signals control reversible program of localization and functional polarization of macrophages.* Cell, 2014. **157**(4): p. 832-44.

58. Molawi, K., et al., *Progressive replacement of embryo-derived cardiac macrophages with age.* J Exp Med, 2014. **211**(11): p. 2151-8.

59. Hume, D.A. and K.P. MacDonald, *Therapeutic applications of macrophage colony-stimulating factor-1 (CSF-1) and antagonists of CSF-1 receptor (CSF-1R) signaling.* Blood, 2012. **119**(8): p. 1810-20.

60. Khosravi, M., et al., *Circulating low density lipoprotein (LDL)*. Horm Mol Biol Clin Investig. 2018 Jul 31;35(2).

61. Frambach SJCM, de Haas R, Smeitink JAM, Rongen GA, Russel FGM, Schirris TJJ. Brothers in Arms: ABCA1- and ABCG1-Mediated Cholesterol Efflux as Promising Targets in Cardiovascular Disease Treatment. Pharmacol Rev. 2020 Jan;72(1):152-190.

62. Besler KJ, Blanchard V, Francis GA. Lysosomal acid lipase deficiency: A rare inherited dyslipidemia but potential ubiquitous factor in the development of atherosclerosis and fatty liver disease. Front Genet. 2022 Sep 20;13:1013266.

63. Lee-Rueckert, M., et al., Acidic extracellular pH promotes accumulation of free cholesterol in human monocyte-derived macrophages via inhibition of ACAT1 activity. Atherosclerosis. 2020 Nov;312:1-7.

64. Hosseni, B., et al., *Plasma PCSK9 level affects passively LAMP-2 expression; an evidence of transcription network.* Gene Reports, 2016. **4**: p. 258-263.

65. Chistiakov, D.A., et al., *Mechanisms of foam cell formation in atherosclerosis.* J Mol Med (Berl), 2017. **95**(11): p. 1153-1165.

66. Thiam AR, Ikonen E. Lipid Droplet Nucleation. Trends Cell Biol. 2021 Feb;31(2):108-118.

67. Koliwad, S.K., et al., *DGAT1-dependent triacylglycerol storage by macrophages protects mice from diet-induced insulin resistance and inflammation.* J Clin Invest, 2010. **120**(3): p. 756-67.

68. Walther, T.C., J. Chung, and R.V. Farese, Jr., *Lipid Droplet Biogenesis.* Annu Rev Cell Dev Biol, 2017. **33**: p. 491-510.

69. Kory, N., R.V. Farese, Jr., and T.C. Walther, *Targeting Fat: Mechanisms of Protein Localization to Lipid Droplets.* Trends Cell Biol, 2016. **26**(7): p. 535-546.

70. Choudhary, V., et al., *A conserved family of proteins facilitates nascent lipid droplet budding from the ER.* J Cell Biol, 2015. **211**(2): p. 261-71.

71. Ben M'barek K, Ajjaji D, Chorlay A, Vanni S, Forêt L, Thiam AR. ER Membrane Phospholipids and Surface Tension Control Cellular Lipid Droplet Formation. Dev Cell. 2017 Jun 19;41(6):591-604.e7.

72. Gao, M., et al., *The biogenesis of lipid droplets: Lipids take center stage.* Prog Lipid Res, 2019. **75**: p. 100989.

73. Hussain SS, Tran TM, Ware TB, Luse MA, Prevost CT, Ferguson AN, Kashatus JA, Hsu KL, Kashatus DF. RalA and PLD1 promote lipid droplet growth in response to nutrient withdrawal. Cell Rep. 2021 Jul 27;36(4):109451.

74. Choudhary, V., et al., *Architecture of Lipid Droplets in Endoplasmic Reticulum Is Determined by Phospholipid Intrinsic Curvature.* Curr Biol, 2018. **28**(6): p. 915-926 e9.

75. Qi, Y., L. Sun, and H. Yang, *Lipid droplet growth and adipocyte development: mechanistically distinct processes connected by phospholipids.* Biochim Biophys Acta Mol Cell Biol Lipids, 2017. **1862**(10 Pt B): p. 1273-1283.

76. Pagac, M., et al., *SEIPIN Regulates Lipid Droplet Expansion and Adipocyte Development by Modulating the Activity of Glycerol-3-phosphate Acyltransferase.* Cell Rep, 2016. **17**(6): p. 1546-1559.

77. Salo, V.T., et al., *Seipin regulates ER-lipid droplet contacts and cargo delivery.* EMBO J, 2016. **35**(24): p. 2699-2716.

78. Jin Y, Tan Y, Zhao P, Ren Z. SEIPIN: A Key Factor for Nuclear Lipid Droplet Generation and Lipid Homeostasis. Int J Mol Sci. 2020 Nov 2;21(21):8208.

79. Cruz, A.L.S., et al., *Lipid droplets: platforms with multiple functions in cancer hallmarks.* Cell Death Dis, 2020. **11**(2): p. 105.

80. Reustle, A. and M. Torzewski, *Role of p38 MAPK in Atherosclerosis and Aortic Valve Sclerosis.* Int J Mol Sci, 2018. **19**(12).

81. Poznyak, A.V., et al., *Signaling Pathways and Key Genes Involved in Regulation of foam Cell Formation in Atherosclerosis.* Cells, 2020. **9**(3).

82. Banerjee, D., et al., *Inflammation-induced mTORC2-Akt-mTORC1 signaling promotes macrophage foam cell formation.* Biochimie, 2018. **151**: p. 139-149.

83. Zheng, H., et al., *mTOR signaling promotes foam cell formation and inhibits foam cell egress through suppressing the SIRT1 signaling pathway.* Mol Med Rep, 2017. **16**(3): p. 3315-3323.

84. Jarc, E. and T. Petan, *Lipid Droplets and the Management of Cellular Stress.* Yale J Biol Med, 2019. **92**(3): p. 435-452.

85. Badimon, L. and G. Vilahur, *Thrombosis formation on atherosclerotic lesions and plaque rupture.* J Intern Med, 2014. **276**(6): p. 618-32.

86. Rekhter, M.D., *Collagen synthesis in atherosclerosis: too much and not enough.* Cardiovasc Res, 1999. **41**(2): p. 376-84.

87. Lopes, J., et al., *Type VIII collagen mediates vessel wall remodeling after arterial injury and fibrous cap formation in atherosclerosis.* Am J Pathol, 2013. **182**(6): p. 2241-53.

88. Bentzon, J.F., et al., *Mechanisms of plaque formation and rupture.* Circ Res, 2014. **114**(12): p. 1852-66.

89. Ulrich, V., et al., *Chronic miR-29 antagonism promotes favorable plaque remodeling in atherosclerotic mice.* EMBO Mol Med, 2016. **8**(6): p. 643-53.

90. Zhu, Y., et al., *Research Progress on the Relationship between Atherosclerosis and Inflammation.* Biomolecules, 2018. **8**(3).

91. Li, P.C., et al., *Anti-Restenotic Roles of Dihydroaustrasulfone Alcohol Involved in Inhibiting PDGF-BB-Stimulated Proliferation and Migration of Vascular Smooth Muscle Cells.* Mar Drugs, 2015. **13**(5): p. 3046-60.

92. Bennett, M.R., S. Sinha, and G.K. Owens, *Vascular Smooth Muscle Cells in Atherosclerosis.* Circ Res, 2016. **118**(4): p. 692-702.

93. Ghasempour, G., et al., *Correlations between vitronectin, miR-520, and miR-34 in patients with stenosis of coronary arteries.* Molecular Biology Reports, 2021. **48**.

94. Francis, A.A. and G.N. Pierce, *An integrated approach for the mechanisms responsible for atherosclerotic plaque regression.* Exp Clin Cardiol, 2011. **16**(3): p. 77-86.

95. Ghasempour, G., et al., *miRNAs through β-ARR2/p-ERK1/2 pathway regulate the VSMC proliferation and migration.* Life Sci, 2021. **279**: p. 119703.

96. Fernández-Hernando, C., et al., *Absence of Akt1 reduces vascular smooth muscle cell migration and survival and induces features of plaque vulnerability and cardiac dysfunction during atherosclerosis.* Arterioscler Thromb Vasc Biol, 2009. **29**(12): p. 2033-40.

97. Colin, S., G. Chinetti-Gbaguidi, and B. Staels, *Macrophage phenotypes in atherosclerosis.* Immunol Rev, 2014. **262**(1): p. 153-66.

98. Huang, X., et al., *Polarizing Macrophages In Vitro.* Methods Mol Biol, 2018. **1784**: p. 119-126.

99. Mohsen, K., et al., *The Increase of pFAK and THBS1 Protein and Gene Expression Levels in Vascular Smooth Muscle Cells by Histamine-treated M1 Macrophages.* Iranian Journal of Allergy, Asthma and Immunology, 2019. **18**(1).

100. Andreou, I., et al., *miRNAs in atherosclerotic plaque initiation, progression, and rupture.* Trends in Molecular Medicine, 2015. **21**(5): p. 307-318.

101. Bobryshev, Y.V., et al., *Macrophages and Their Role in Atherosclerosis: Pathophysiology and Transcriptome Analysis.* Biomed Res Int, 2016. **2016**: p. 9582430.

102. Jain, N., J. Moeller, and V. Vogel, *Mechanobiology of Macrophages: How Physical Factors Coregulate Macrophage Plasticity and Phagocytosis.* Annual Review of Biomedical Engineering, 2019. **21**(1): p. 267-297.

103. Ylä-Herttuala, S., et al., *Stabilization of atherosclerotic plaques: an update.* Eur Heart J, 2013. **34**(42): p. 3251-8.

104. Yarnazari, A., et al., *The sdLDL Reduces MRC1 Expression Level and Secretion of Histamin e in Differentiated M2-macrophages from Patients with Coronary Artery Stenosis.* Cardiovasc Hematol Disord Drug Targets, 2017. **17**(1): p. 28-32.

105. Lloyd, A.F. and V.E. Miron, *Cellular and Molecular Mechanisms Underpinning Macrophage Activation during Remyelination.* Front Cell Dev Biol, 2016. **4**: p. 60.

106. Moore, K.J., F.J. Sheedy, and E.A. Fisher, *Macrophages in atherosclerosis: a dynamic balance.* Nat Rev Immunol, 2013. **13**(10): p. 709-21.

107. Vergallo, R. and F. Crea, *Atherosclerotic Plaque Healing.* N Engl J Med, 2020. **383**(9): p. 846-857.

108. Poznyak, A.V., et al., *Oxidative Stress and Antioxidants in Atherosclerosis Development and Treatment.* Biology (Basel), 2020. **9**(3).

109. Insull, W., Jr., *The pathology of atherosclerosis: plaque development and plaque responses to medical treatment.* Am J Med, 2009. **122**(1 Suppl): p. S3-s14.

110. Hermus, L., et al., *Carotid plaque formation and serum biomarkers.* Atherosclerosis, 2010. **213**(1): p. 21-29.

111. Chaher, N., et al., *Imaging the Extracellular Matrix in Prevalent Cardiovascular Diseases.* Applied Sciences, 2020. **10**(11).

112. Petsophonsakul, P., et al., *Role of Vascular Smooth Muscle Cell Phenotypic Switching and Calcification in Aortic Aneurysm Formation.* Arterioscler Thromb Vasc Biol, 2019. **39**(7): p. 1351-1368.

113. Kowara, M. and A. Cudnoch-Jedrzejewska, *Pathophysiology of Atherosclerotic Plaque Development-Contemporary Experience and New Directions in Research.* Int J Mol Sci, 2021. **22**(7).

114. Sterpetti, A.V., *Inflammatory Cytokines and Atherosclerotic Plaque Progression. Therapeutic Implications.* Curr Atheroscler Rep, 2020. **22**(12): p. 75.

115. Fortini, C., et al., *The role of the Notch pathway in atherosclerosis.* Indian Journal of Cardio Biology & Clinical Sciences, 2014. **1**(1): p. 103.

116. Andreou, I., et al., *miRNAs in atherosclerotic plaque initiation, progression, and rupture.* Trends Mol Med, 2015. **21**(5): p. 307-18.

117. Tabas, I., *Macrophage apoptosis in atherosclerosis: consequences on plaque progression and the role of endoplasmic reticulum stress.* Antioxid Redox Signal, 2009. **11**(9): p. 2333-9.

118. Tabas, I., G. García-Cardeña, and G.K. Owens, *Recent insights into the cellular biology of atherosclerosis.* J Cell Biol, 2015. **209**(1): p. 13-22.

119. Theodorou, K. and R.A. Boon, *Endothelial Cell Metabolism in Atherosclerosis.* Front Cell Dev Biol, 2018. **6**: p. 82.

120. Dekker, R.J., et al., *KLF2 provokes a gene expression pattern that establishes functional quiescent differentiation of the endothelium.* Blood, 2006. **107**(11): p. 4354-63.

121. Doddaballapur, A., et al., *Laminar shear stress inhibits endothelial cell metabolism via KLF2-mediated repression of PFKFB3.* Arterioscler Thromb Vasc Biol, 2015. **35**(1): p. 137-45.

122. Feng, S., et al., *Mechanical Activation of Hypoxia-Inducible Factor 1α Drives Endothelial Dysfunction at Atheroprone Sites.* Arterioscler Thromb Vasc Biol, 2017. **37**(11): p. 2087-2101.

123. Wu, D., et al., *HIF-1α is required for disturbed flow-induced metabolic reprogramming in human and porcine vascular endothelium.* eLife, 2017. **6**: p. e25217.

124. Xu, H., et al., *Vascular Macrophages in Atherosclerosis.* J Immunol Res, 2019. **2019**: p. 4354786.

125. Čejková, S., I. Králová-Lesná, and R. Poledne, *Monocyte adhesion to the endothelium is an initial stage of atherosclerosis development.* Cor et Vasa, 2016. **58**(4): p. e419-e425.

126. Schrijvers, D.M., et al., *Phagocytosis in atherosclerosis: Molecular mechanisms and implications for plaque progression and stability.* Cardiovasc Res, 2007. **73**(3): p. 470-80.

127. Lillis, A.P., et al., *LDL Receptor-Related Protein-1 (LRP1) Regulates Cholesterol Accumulation in Macrophages.* PLoS One, 2015. **10**(6): p. e0128903.

128. Perrey, S., et al., *Preferential pharmacological inhibition of macrophage ACAT increases plaque formation in mouse and rabbit models of atherogenesis.* Atherosclerosis, 2001. **155**(2): p. 359-370.

129. Pan, J., et al., *Role of vascular smooth muscle cell phenotypic switching in plaque progression: A hybrid modeling study.* Journal of Theoretical Biology, 2021. **526**: p. 110794.

130. Stehbens, W.E., *Cerebral atherosclerosis. Intimal proliferation and atherosclerosis in the cerebral arteries.* Arch Pathol, 1975. **99**(11): p. 582-91.

131. Ghasempour, G., et al., *Upregulation of TGF-β type II receptor in high glucose-induced vascular smooth muscle cells.* Molecular Biology Reports, 2022. **49**(4): p. 2869-2875.

132. Doran, A.C., N. Meller, and C.A. McNamara, *Role of smooth muscle cells in the initiation and early progression of atherosclerosis.* Arterioscler Thromb Vasc Biol, 2008. **28**(5): p. 812-9.

133. Wang, Z., D. Wang, and Y. Wang, *Cigarette Smoking and Adipose Tissue: The Emerging Role in Progression of Atherosclerosis.* Mediators of Inflammation, 2017. **2017**: p. 3102737.

134. Clarke, M. and M. Bennett, *The Emerging Role of Vascular Smooth Muscle Cell Apoptosis in Atherosclerosis and Plaque Stability.* American Journal of Nephrology, 2006. **26**(6): p. 531-535.

135. Morris, P.B., et al., *Cardiovascular Effects of Exposure to Cigarette Smoke and Electronic Cigarettes: Clinical Perspectives From the Prevention of Cardiovascular Disease Section Leadership Council and Early Career Councils of the American College of Cardiology.* J Am Coll Cardiol, 2015. **66**(12): p. 1378-91.

136. Holme, J.A., et al., *Potential role of polycyclic aromatic hydrocarbons as mediators of cardiovascular effects from combustion particles.* Environ Health, 2019. **18**(1): p. 74.

137. Shami, A., I. Gonçalves, and A. Hultgårdh-Nilsson, *Collagen and related extracellular matrix proteins in atherosclerotic plaque development.* Curr Opin Lipidol, 2014. **25**(5): p. 394-9.

138. Chiorescu RM, Mocan M, Inceu AI, Buda AP, Blendea D, Vlaicu SI. [Vulnerable Atherosclerotic Plaque: Is There a Molecular Signature?](https://pubmed.ncbi.nlm.nih.gov/36362423/) Int J Mol Sci. 2022 Nov 7;23(21):13638. doi: 10.3390/ijms232113638.

139. Noothi SK, Ahmed MR, Agrawal DK. [Residual risks and evolving atherosclerotic plaques.](https://pubmed.ncbi.nlm.nih.gov/36897542/) Mol Cell Biochem. 2023 Mar 10. doi: 10.1007/s11010-023-04689-0. Online ahead of print.

140. Silvestre-Roig, C., et al., *Atherosclerotic plaque destabilization: mechanisms, models, and therapeutic strategies.* Circulation research, 2014. **114**(1): p. 214-226.

141. Quillard, T., et al., *Mechanisms of erosion of atherosclerotic plaques.* Curr Opin Lipidol, 2017. **28**(5): p. 434-441.

142. Yurdagul A Jr. Crosstalk Between Macrophages and Vascular Smooth Muscle Cells in Atherosclerotic Plaque Stability. Arterioscler Thromb Vasc Biol. 2022 Apr;42(4):372-380.

143. Arai T, Sekimoto T, Koba S, Mori H, Matsukawa N, Sakai R, Yokota Y, Sato S, Tanaka H, Masaki R, Oishi Y, Ogura K, Arai K, Nomura K, Sakai K, Tsujita H, Kondo S, Tsukamoto S, Suzuki H, Shinke T. [Impact of small dense low-density lipoprotein cholesterol and triglyceride-rich lipoproteins on plaque rupture with ST-segment elevation myocardial infarction.](https://pubmed.ncbi.nlm.nih.gov/36038471/) J Clin Lipidol. 2022 Sep-Oct;16(5):725-732.

144. Sakakura, K., et al., *Pathophysiology of Atherosclerosis Plaque Progression.* Heart, Lung and Circulation, 2013. **22**(6): p. 399-411.

145. Luo X, Lv Y, Bai X, Qi J, Weng X, Liu S, Bao X, Jia H, Yu B. Plaque Erosion: A Distinctive Pathological Mechanism of Acute Coronary Syndrome. Front Cardiovasc Med. 2021 Sep 28;8:711453. doi: 10.3389/fcvm.2021.711453. eCollection 2021.

146. Camaré, C., et al., *Angiogenesis in the atherosclerotic plaque.* Redox Biol, 2017. **12**: p. 18-34.

147. Falk, E., *Pathogenesis of atherosclerosis.* J Am Coll Cardiol, 2006. **47**(8 Suppl): p. C7-12.

148. Tarkin, J.M., et al., *Imaging Atherosclerosis.* Circ Res, 2016. **118**(4): p. 750-69.

149. Stoll, G. and M. Bendszus, *Inflammation and atherosclerosis: novel insights into plaque formation and destabilization.* Stroke, 2006. **37**(7): p. 1923-32.

150. Firoozrai, M., et al., *Angiotensin Converting Enzyme(ACE) Activity, Levels of Lipids and Apolipoproteins in Patients with Coronary Artery Disease.* RJMS, 2008. **14**(57): p. 151-156.

151. de Boer, O.J., A.C. van der Wal, and A.E. Becker, *Atherosclerosis, inflammation, and infection.* The Journal of pathology, 2000. **190**(3): p. 237-243.

152. Xu, S., J. Pelisek, and Z.G. Jin, *Atherosclerosis Is an Epigenetic Disease.* Trends Endocrinol Metab, 2018. **29**(11): p. 739-742.

153. Bürrig, K.F., *The endothelium of advanced arteriosclerotic plaques in humans.* Arteriosclerosis and thrombosis: a journal of vascular biology, 1991. **11**(6): p. 1678-1689.

154. Penn, A. and C. Snyder, *Arteriosclerotic plaque development is ‘promoted’ by polynuclear aromatic hydrocarbons.* Carcinogenesis, 1988. **9**(12): p. 2185-2189.

155. Meyer, T., et al., *Expression of meta-vinculin in human coronary arteriosclerosis is related to the histological grade of plaque formation.* Atherosclerosis, 1994. **111**(1): p. 111-9.

Figure 1: Leukocyte paracellular and transcellular diapedesis ways. The roles of adhesion molecules on Capture (1), Rolling (2), Adhesion (3) and Crawling (4) of leukocytes. Some genes involved in transcellular (5.A) and paracellular (5.B) ways. BioRender.com.

Figure 2: Polarization of monocyte to M3 macrophage. BioRender.com.

Figure 3: Polarization of monocyte to M4 macrophage. BioRender.com.

Figure 4: Polarization of monocyte to M2 macrophage. Several signaling pathways contribute to polarize the different forms of M2 macrophage such as M2a, M2b and M2c. BioRender.com.

Figure 5: Polarization of monocyte to M1 macrophage. BioRender.com.

Figure 6: Formation of fat droplets in cell. The modified LDL particles are transferred by membrane receptors (1) into endosomes (2, 3). The primary structures including cholesteryl esters (CE) originated from lysosomes and cytosol (4) are produced on endoplasmic reticulum (5). Finally, fat droplets (6) are produced in cytosol. BioRender.com.

Figure 7: Atherosclerotic plaque formation and progression. (A), The plaque is developed during the different steps including the leukocyte diapedesis. (B), Differentiation and polarization. (C), VSMCs proliferation and migration and (D), plaque remodeling. BioRender.com.

Figure 8: Plaque rupture and erosion. The mature plaque (A) may be Rupture (B) or Erosion (C). BioRender.com.
